# Social accountability and sexual and reproductive health—implications for research and practice

**DOI:** 10.1186/s12939-023-02081-4

**Published:** 2024-01-08

**Authors:** Petrus S. Steyn, Victoria Boydell, Sana Contractor, Joanna Paula Cordero, Ana Lorena Ruano

**Affiliations:** 1 UNDP/UNFPA/UNICEF/WHO/World Bank Special Programme of Research, Development of Sexual and Research Training in Human Reproduction (HRP Research), Department of Sexual and Reproductive Health and Research, World Health Organization, Geneva, Switzerland; 2 Institute of Women’s Health, University College London, London, UK; 3 Centre for International Health, Institute of Tropical Medicine Antwerp, University of Bergen Norway, Antwerp, Belgium; 4 COPASAH, New Delhi, India; 5 The George Institute for Global Health New Delhi, New Delhi, India; 6 Center for International Health, Department of Global Public Health & Primary Care, University of Bergen, Bergen, Norway

This supplement entitled “Social accountability and sexual and reproductive health—Implications for Research and Practice” in the *International Journal for Equity in Health* journal responds to some of the ongoing challenges in researching social accountability in the context of sexual and reproductive health, rights and justice. Social accountability, which is defined as “citizens’ efforts at ongoing meaningful collective engagement with public institutions for accountability in the provision of public goods” [[1], p161] is gaining increasing attention in SRHR [2].

Accountability is a complex process. Accountability processes feature multiple and interrelated components, steps, and actors, with several simultaneous processes of change, triggering collective changes [3]. Social accountability is relational and focuses on transforming relationships between communities and health systems, but also between actors within these two systems at different levels; therefore, interventions change and adapt as they are rolled out in specific political and social contexts. Descriptive and analytic work on implementation is further complicated by the broad and interrelated set of outcomes that together facilitate change. Outcomes may include both routine health and service delivery outcomes [4], alongside changes in self-efficacy, social cohesion, trust, and responsiveness [5]. Even when the outcomes are delineated, it may be difficult to attribute them to a specific part of a multi-component intervention. Given this complexity, researching social accountability in the context of sexual and reproductive health, rights and justice is complicated.

Research in this area is fast evolving. Recent research in the field encompasses more than directly measurable health-related outcomes and now includes a wider range of governance outcomes such as empowerment, participation, and the responsiveness of duty-bearers [6]. Yet evaluating a broader range of interrelated outcomes poses several methodological challenges [7, 8]. Research exploring how social accountability interventions related to health achieve their effects continue to increase [7]. There is also an increasing awareness of the centrality of power dynamics in social accountability process and the need to use research methodologies that account for them [6]. Despite these advances, gaps still remain. Marston et al., found social accountability studies remain limited when collecting data and analysing the wider context such as sociopolitical dimensions that may illuminate processes and allow for transferability of interventions [7]. Additionally, none of the studies that they reviewed reported on whether there financial dependencies between the evaluators and the implementers that may have an effect on the evaluation [7].

To help address some of these continuing challenges, this supplement brings together different learnings to advance researching social accountability in the context of sexual and reproductive health, rights and justice. This covers both learning from research, from practice and wider theoretical contributions.

## Learnings from research

In this supplement, three articles reflect on the current state of research and detail the means through which they address challenges in studying or evaluating social accountability. Specifically, the articles explore what research questions to evaluate, how to conduct social accountability research and how to report on them.

Van Belle details how realist evaluation can be used to answer not only questions of effectiveness but also the causal questions ‘how, why, in which conditions and for whom’ [9]. Van Belle shares a protocol of a proposed study that use different social science methods to explore the effect of multi-level governance arrangements on sexual and reproductive health of adolescents living in informal settlements in Mumbai, Delhi, Cotonou and Kampala.

Kraft et al. identified reporting gaps in social accountability research through a review of reviews and proposed a checklist based on CONSORT SPI to help researchers full report their studies [10]. Social accountability studies tend to have shortcomings in reporting on conceptual underpinnings; site description; study information; intervention; context; study design; outcomes; and analyses. The final Social Accountability Reporting for Research (SAR4Research) aims to help ensure that results of social accountability evaluations in peer-reviewed literature will be more useful, facilitating learning and application of findings, when study designs, interventions and their context are described fully in one or a set of papers.

The article by Cordero et al. reflects on the interaction between research and implementation components in a multi-country study evaluating the effect of social accountability on contraceptive services [11]. The article details how the Community and Provider driven Social Accountability Intervention (CaPSAI) study operationalized the Medical Research Council (MRC) guidance on process evaluation of a complex intervention [12] and make the case for social accountability research to include clear statements explaining the nature and types of relationships between researchers and implementers involved in the intervention.

## Learnings from practice

Three manuscripts in this supplement have been written by practitioner-researchers, and they throw light on the rich, multi-layered tapestry that constitutes accountability practice in SRHR. The article by Robinson and Adams [13] describes the process and outcomes of a strategic accountability campaign to improve maternal health in Niger State of Nigeria, tracing its journey from first building a demand for accountability through mobilizing citizens, articulating demands, and organizing public dialogues, followed by a second phase in which it attempted to strengthen accountability mechanisms. The authors reflect on the successes and the challenges faced at each stage by this nascent accountability initiative, highlighting the complexity of the problem, as well as the need for consistent long-term engagement to address it.

Goswami and Pinto’s article, on the other hand, describes the process of integrating maternal health as an issue of concern within a larger campaign for rights and dignity by Dalit women in Karnataka State in India [14]. They foreground accountability efforts to improve maternal health, within the long-standing efforts to “*conscientize-organize-struggle*” (drawing on Dalit liberation leader Dr. B.R.Ambedkar’s philosophy), highlighting the importance of conceptualizing the right to maternal health within the larger framework of Dalit women’s struggle for dignity and against structural oppression.

Finally, Bailey and Mujune’s contribution describes an “induced intervention” – Accountability Can Transform Health (or ACT Health) – in Uganda which, in its first phase had been evaluated using an RCT and found to have little or no impact on health outcomes [15]. In this paper, the authors describe the second phase of the intervention, which replaced the earlier light-touch facilitation with strict parameters (as required by the RCT design), with guided, iterative strategic practice and more continuous program monitoring. In contrast to the experimental designs which restrict both interventions and analysis, the authors use the data from this second phase to highlight the value that research methodologies utilizing monitoring or process data are able to bring to the study of un-even, geographically diverse, and iterative interventions, with differing outcomes at multiple levels. Collectively, the three practice papers in this issue, include a diversity of interventions and research methodologies, and draw attention to the need for more creativity and innovation in how accountability interventions are designed and studied. Most importantly, they highlight the value of research conducted by practitioners themselves rather than by experts alone.

### Theoretical contributions

Two commentaries in this supplement share the collective reflections of both practitioners and researchers and identify some theoretical challenges in the study of strengthening social accountability practices. Accountability is both a means and an end of rights-based approaches, which can lead to certain dissonances in our understanding. Social accountability as an end of a rights-based approach, e.g., the right to accountability, can often mask inequities and differences in accountability practices themselves; for example, deliberatively building consensus can result in the needs of a few being sidelined for the majority.

Schaaf et al., in this volume, remind us that we cannot assume that social accountability processes are automatically inclusive of ‘excluded and historically oppressed populations” [16]. Stigma, and harmful gender norms among providers, local social and political hierarchies and communities, and lack of guidance and knowledge of SRHR entitlement can limit fully inclusive processes. The commentary by Schaaf et al. describes several strategies employed by programme implementers to engage excluded populations actively. These programmatic experiences synthesize some principles for ensuring that social accountability efforts are inclusive in terms of people included in the process and issues being addressed.

In a similar vein, using a historical lens, Nelson et al., in this volume, outline two broad approaches to social accountability programmes, the ‘technicians” and the ‘activists’, that indicate different means of ensuring social accountability [17]. These two approaches to supporting social accountability emerge from a deep-rooted tension in international health between long and short-term approaches. Naming these two schools of thought in social accountability and detailing their respective relationships to government, to communities, and setting out different goals, technicity and resources help to understand better how social accountability is achieved and what needs. The ‘technicians’ have a more instrumental approach to addressing failures in the health system, whereas the activists focus on transforming the underlying structural barriers. A conciliatory conclusion is to accept the plurality of perspectives and actively recognize the legitimacy of agendas and approaches and this “openness and a willingness to be made uncomfortable when confronted with the limitations of one’s own assumptions”. Finding this ‘middle ground’ is a contested learning process.

In the field of sexual and reproductive health rights, where there is an ever-present contestation between the aspirational framework of human rights, and lived socio-political realities that constrain their realisation, efforts to seek, enforce or demand accountability are invaluable. The papers in this supplement cover a range of such efforts, implemented in varied, dynamic contexts which further emphasize the complexity of knowledge creation on this subject. They repeatedly highlight the interconnectedness of SRHR with not just the health system but also social and political systems, especially in the case of excluded and marginalized groups. At the same time, they provide new creative methodologies and important theoretical insights, that we hope adds to the growing body of sophisticated and nuanced research.

## Data Availability

Not applicable.

## Abbreviations

SRHR: Sexual and Reproductive Health and Rights
